# Characterization of transferable antibiotic resistance plasmids in airborne particulate matter from ICU environments

**DOI:** 10.1016/j.isci.2025.112254

**Published:** 2025-03-20

**Authors:** Kexing Zhang, Xumei Zhou, Xu Zhang, Na Huang, Zhengyang Zhao, Xinqiang Zhang, Yong zhou, Juntao Li, Fangyi Yu, Yuan Liu, Pengzhe Qin, Xinwei Wu, Peng He

**Affiliations:** 1 School of Public Health, Sun Yat-sen University, Guangzhou, Guangdong 510080, P.R. China; 2 Guizhou Hospital, The First Affiliated Hospital of Sun Yat-sen University, Guiyang, Guizhou 550031, P.R. China; 3 Guangzhou Center for Disease Control and Prevention (Guangzhou Health Supervision institute), Guangzhou, Guangdong 510440, P.R. China; 4 Department of Healthcare-associated Infection Management, Guangzhou Red Cross Hospital, Guangzhou, Guangdong 510220, P.R. China; 5 Institute of Public Health, Guangzhou Medical University & Guangzhou Center for Disease Control and Prevention, Guangzhou, Guangdong 510440, P.R. China

**Keywords:** Health sciences, Medicine, Medical specialty, Internal medicine, Medical microbiology, Natural sciences, Biological sciences, Microbiology

## Abstract

Intensive care units (ICUs) are critical environments for the emergence of antibiotic-resistant bacteria, with numerous studies focusing on resistant pathogens in these settings. However, transferable antibiotic resistance plasmids (TARPs)—regardless of their origin from pathogenic or non-pathogenic bacteria—are key drivers of resistance gene dissemination and the emergence of resistant strains. This study investigated TARPs in ICU air. Air samples were directly used to isolate resistant plasmids using *Escherichia coli* CV601 as the recipient. Plasmid types, antibiotic resistance genes (ARGs), and virulence factors were identified through sequencing, and resistance phenotypes were validated. A total of 30 distinct plasmid types were detected, with IncX3 being the most prevalent. Among 245 ARGs identified, *bla*_NDM-53_, *bla*_SHV-12_, and *BRP(MBL)* were dominant. Phylogenetic analysis indicated that these TARPs originated from bacteria commonly colonizing human mucosa. ICU airborne TARPs may significantly contribute to the spread of ARGs and antibiotic resistance transmission.

## Highlights

• Transferable antibiotic resistance plasmids in airborne particles from ICU were investigated
• A total of 30 plasmid types and 47 subtypes of antibiotic resistance genes were identified
• IncX3 plasmids were detected in ICU air for the first time
• The *bla*_NDM-1_ and *bla*_NDM-53_ resistance genes were discovered in ICU air for the first time

## Graphical abstract

**Figure:**
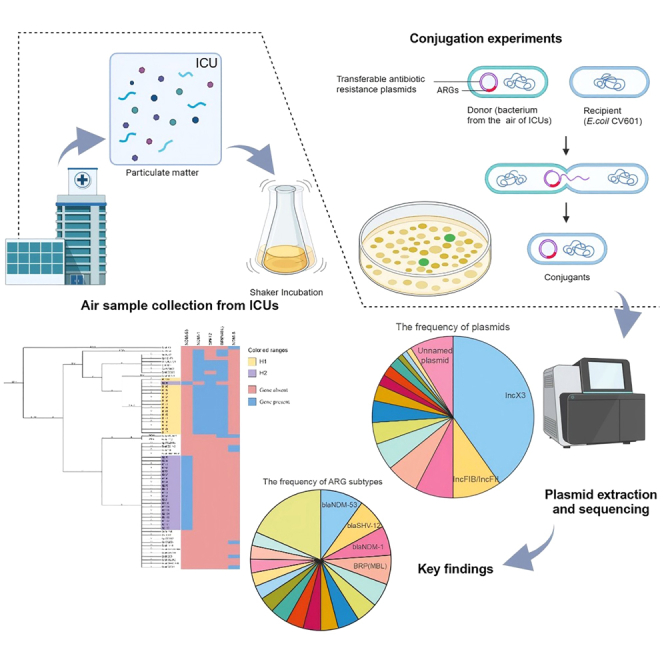

## Introduction

Hospital-acquired infections (HAIs), also known as nosocomial infections (NIs), are a major cause of morbidity and mortality among hospitalized patients, contributing to increased healthcare costs and prolonged hospital stays in both developed and developing countries.^1^ HAIs are a significant public health issue worldwide, with a prevalence of approximately 5%–10% in industrialized countries and about 20%–25% in developing countries.^2^ The highest rates of HAIs are reported in ICUs. A study involving 1,265 ICUs across 76 countries found that 51% of patients were affected.^3^ While numerous studies have demonstrated that drug-resistant bacteria on ICU surfaces, in sewage, and among healthcare workers are significant contributors,^4^^,^^5^^,^^6^^,^^7^ the role of airborne transmission has received comparatively less attention. Unexplained HAIs remain frequent, and air may be a significant transmission route.

Although many studies have reported the presence of airborne antibiotic resistance genes (ARGs), investigations specifically addressing their occurrence and impact within hospital environments, particularly ICUs, are limited. Studies have shown that most patients with methicillin-resistant *Staphylococcus aureus* (MRSA) infection or colonization of the respiratory tract shed viable MRSA into the air of their room.^8^ Yakupogullari et al. demonstrated the presence of *Acinetobacter baumannii* strains from previously infected patients in hospital air that subsequently infected hospitalized patients 3 months later.^9^ These findings highlight the potential for airborne ARGs to contribute to HAI dissemination in ICUs, emphasizing the need for further research into their characteristics, mechanisms, and clinical implications. A deeper understanding of the characteristics of plasmid-borne ARG transmission in ICUs will be crucial for devising targeted infection control strategies and mitigating the risks of HAIs.

In recent years, the bacterial resistance to antibiotics in hospital environments has significantly increased.^10^ Airborne MRSA and methicillin-resistant coagulase-negative *Staphylococcus* have been detected and isolated in various clinical wards such as ICUs, operating rooms, and organ transplant departments.^11^^,^^12^ In fact, few bacteria are naturally resistant to antibiotics. These resistance genes can be transferred between bacteria via mobile genetic elements such as plasmids, integrons, and transposons, and can spread between both pathogenic and non-pathogenic bacteria.^13^^,^^14^ Plasmids are recognized as important vehicles for the horizontal transfer of ARGs.^14^ Once acquired by bacteria, ARGs can lead to the emergence of multidrug-resistant (MDR), extensively drug-resistant (EDR), and even pandrug-resistant (PDR) strains.^15^ The emergence of antimicrobial resistance in clinical pathogens is frequently linked to plasmid activity. For example, the plasmid-borne carbapenem resistance genes *bla*_KPC_ and *bla*_OXA-48_, as well as the colistin resistance gene *mcr*, are increasingly spreading among clinical pathogens under the selective pressure of antibiotics.^16^^,^^17^^,^^18^ Understanding antimicrobial resistance in hospital environments and analyzing the role of mobile genetic elements, such as plasmids, in facilitating the transfer and dissemination of resistance genes are critical for the prevention and control of HAIs. Most studies focus primarily on resistance plasmids in pathogenic bacteria, often neglecting the transferable plasmids carried by non-pathogenic bacteria. However, it is the transferable plasmids—whether from pathogenic or non-pathogenic bacteria—that play a crucial role in driving the spread of antibiotic resistance and should therefore be the central focus of our efforts.

Previous studies predominantly used traditional methods such as polymerase chain reaction (PCR) and multilocus sequence typing (MLST) to identify antibiotic resistance and virulence genes in drug-resistant plasmids. However, these methods could only confirm the presence of specific genes without providing details on plasmid types or gene locations. Whole genome sequencing (WGS) offers a more comprehensive approach, identifying all relevant genes and enabling more accurate typing and variation analysis through comparison with public databases.^19^

In this study, we focus on the transferable antibiotic resistance plasmids (TARPs) in airborne particulate matter (PM) from ICU. To elucidate their characteristics, the airborne particles were sampled in ICUs, then conjugation experiments were performed. The 2nd short-read and 3rd nanopore long-read sequencing were used to sequencing the plasmids of conjugants. After assembly, the plasmid types, resistance genes, and virulence factors were identified. The evolutionary relationships of the plasmids were also analyzed. Finally, minimum inhibitory concentration (MIC) assays were performed on the transconjugants to confirm their resistance, and the correlation between phenotype and genotype was further examined.

## Results

### Bacterial conjugants

A total of 49 samples of ICU air were collected. The samples were collected from 13 hospitals include 12 tertiary hospitals and 1 first-level hospital. We collected data on the number of ICU beds; disinfection measures; frequencies for air, surfaces, and floors; ICU type; and patient types across different hospitals (Table S1). However, our study found that the presence of antibiotic resistance plasmids in the air was not correlated with these factors.

The positive samples were distributed across 5 hospitals, with a conjugation success rate of 20.41% (10/49). According to the morphology and size of the colonies, a total of 57 antibiotic resistance conjugants were isolated from ICU air samples through conjugation experiments (Table 1).

**Table 1:** Distribution of positive samples and positive strains

Hospital	District	Positive samples	Antibiotics resistance	Positive strains
H1	Yuexiu	3	CIP, CTX, CLA, GEN, CFT	24
H2	Yuexiu	3	MEM, CAZ, CFT	24
H3	Tianhe	2	CLA	6
H4	Baiyun	1	CLA, TGC	1
H5	Tianhe	1	GEN	2
Total		10		57

Positive sample: Refers to ICU air samples that successfully conjugated.

Positive strain: Refers to the number of transconjugants obtained from the positive samples.

Antibiotics resistance: Refers to the resistance demonstrated by the transconjugants on the respective three-antibiotic plates.

### Drug susceptibility of conjugants

**Figure 1:**
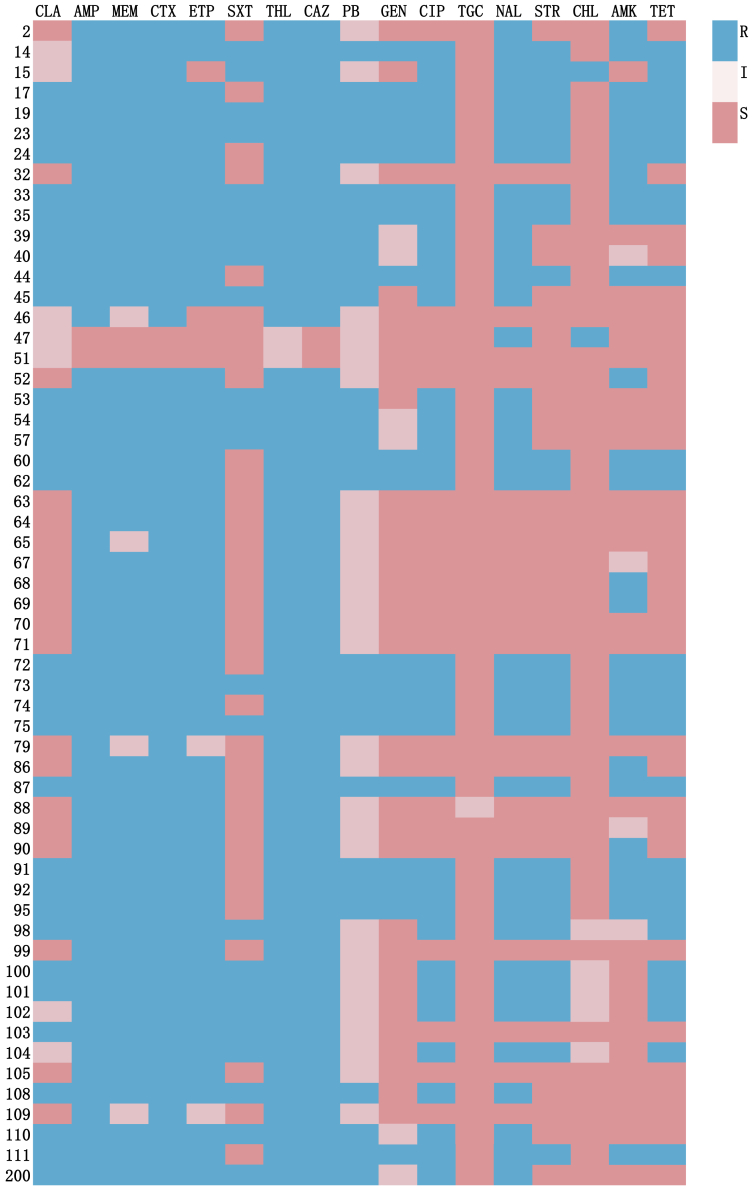
Results of MIC tests of conjugants from air of ICU S, sensitive; I, intermediate; R, resistant; CLA, clarithromycin; AMP, ampicillin; MEM, meropenem; CTX, cefotaxime; ETP, ertapenem; SXT, cotrimoxazole; CFT, cefotiam; CAZ, ceftazidime; PB, polymyxin B; GEN, gentamicin; CIP, ciprofloxacin; TGC, tigecycline; NAL, nalidixic acid; STR, streptomycin; CHL, chloramphenicol; AMK, amikacin; TET, tetracycline.

To validate the antibiotic resistance of transferable plasmids, a total of 57 transconjugants from ICU air were successfully recovered and subjected to antimicrobial susceptibility testing (Table 2). The four antibiotics with the highest resistance rates among these transconjugants were ampicillin (AMP), cefotaxime (CTX), cefotiam (CFT), and ceftazidime (CAZ), each exhibiting resistance in 96.49% (55/57) of the samples (Figure 1). This indicates that these antibiotics demonstrate high resistance in over 90% of the bacterial isolates, reflecting relatively low antibacterial efficacy.

**Table 2:** Results of MIC tests of conjugants from air of ICU

Antibiotic	S (%)	I (%)	R (%)
CLA	19 (35.19)	7 (12.96)	31 (54.38%)
AMP	2 (3.70)	0	55 (96.49%)
MEM	2 (3.70)	4 (7.41)	51 (89.47%)
CTX	2 (3.70)	0	55 (96.49%)
ETP	3 (5.56)	3 (5.56)	51 (89.47%)
SXT	33 (61.11)	0	23 (40.35%)
CFT	0	2 (3.70)	55 (96.49%)
CAZ	2 (3.70)	0	55 (96.49%)
PB	0	29 (53.70)	28 (49.12%)
GEN	30 (55.56)	6 (11.11)	19 (33.33%)
CIP	23 (42.59)	0	34 (59.64%)
TGC	53 (98.15)	0	0 (0.00%)
NAL	21 (38.89)	0	36 (63.15%)
STR	30 (55.56)	0	25 (43.85%)
CHL	47 (87.04)	5 (9.26)	2 (0.35%)
AMK	25 (46.30)	4 (7.40)	26 (45.61%)
TET	30 (55.56)	0	25 (43.85%)

S, sensitive; I, intermediate; R, resistant; CLA, clarithromycin; AMP, ampicillin; MEM, meropenem; CTX, cefotaxime; ETP, ertapenem; SXT, cotrimoxazole; CFT, cefotiam; CAZ, ceftazidime; PB, polymyxin B; GEN, gentamicin; CIP, ciprofloxacin; TGC, tigecycline; NAL, nalidixic acid; STR, streptomycin; CHL, chloramphenicol; AMK, amikacin; TET, tetracycline.

Notably, 96.49% (55/57) of the transconjugants showed resistance to four or more antibiotics. Among these, six transconjugants were resistant to 15 antibiotics, 13 transconjugants were resistant to 14 antibiotics, three transconjugants were resistant to 12 antibiotics, and 12 transconjugants were resistant to 11 antibiotics.

### Plasmid categories

Out of 57 antibiotic resistance conjugants isolated from ICU air and TARPs were found in 80.70% (46/57) of conjugants. Thirty different plasmid types were identified, with IncX3 being the most prevalent (80.43%, 37/46), followed by IncFIB/IncFII at 19.57% (9/46) (Table 3).

**Table 3:** The frequency of plasmids

Type of plasmids	Number of positive strains	Frequency (%)
IncX3	37	80.43%
IncFIB/IncFII	9	19.57%
rep_cluster_2335	7	15.22%
rep_cluster_1968	6	13.04%
rep_cluster_867	5	10.87%
rep_cluster_1291	4	8.70%
rep_cluster_1197	4	8.70%
rep_cluster_2335/rep_cluster_2358	3	6.52%
rep_cluster_481	2	4.35%
IncFII	2	4.35%
IncFIB	2	4.35%
rep_cluster_185	1	2.17%
ColRNAI_rep_cluster_1987	1	2.17%
ColRNAI_rep_cluster_1857	1	2.17%
Unnamed plasmid	8	17.39%

Type of plasmids was annotated using PlasmidFinder or Mob_recon

Number of positive strains: Indicates the number of transconjugants in which this type of plasmid was detected.

### The antibiotic resistance genes carried on plasmids of conjugants

We identified a total of 245 resistance genes on the conjugative plasmids, classified into 47 subtypes, that conferred resistance to 17 drug classes of antibiotics based on Comprehensive Antibiotic Research Database (CARD) (Table 4). Of these, 144 were genes conferring resistance to only one drug class, and 101 conferred resistances to multiple drug classes. Genes conferring resistance to only one drug class include aminoglycoside ARGs *APH(3″)-Ib* and *APH(6)-Id* (11/46, 23.91%), *AAC(6′)-Ib10* (9/46, 19.57%) and *AAC(3)-IIe* (7/46, 15.22%), tetracycline (TET) ARG *tet(A)* (7/46, 15.22%), glycopeptide ARGs *BRP(MBL)* (15/46, 32.61%), fluoroquinolone ARGs *QnrS1* (8/46, 17.39%), *QnrB1* (7/46, 14.89%), macrolide ARGs *macB* (3/46, 6.52%), *mphE* (2/46, 4.35%), sulfonamide ARGs *sul2* (12/46, 26.09%), *sul1* (2/46, 4.35%), and diaminopyrimidine antibiotic gene *dfrA14* (7/46, 14.89%). Genes conferring resistance to multiple drug classes include *bla*_NDM-53_ (22/46, 47.83%) and *bla*_NDM-1_ (15/46, 32.61%), both of which cf. resistance to carbapenem, cephalosporin, cephamycin, and penam antibiotic. The ***bla***_SHV-12_ gene (16/46, 34.78%) was also prevalent, conferring resistance to cephalosporins and penams, while ***bla***_TEM-1_ (9/46, 19.57%) conferred resistance to monobactams, cephalosporins, penams, and penems.

**Table 4:** The frequency of ARG subtypes

Category of ARGs	Number of positive samples (*n* = 46)	Frequency (%)	Drug class
*bla*_*NDM-53*_	22	47.83%	carbapenem; cephalosporin; cephamycin; penam
*bla*_SHV-12_	16	34.78%	cephalosporin; penam
*bla*_NDM-1_	15	32.61%	carbapenem; cephalosporin; cephamycin; penam
*BRP(MBL)*	15	32.61%	glycopeptide antibiotic
*sul2*	12	26.09%	sulfonamide antibiotic
*APH(6)-Id*	11	23.91%	aminoglycoside antibiotic
*APH(3″)-Ib*	11	23.91%	
*bla*_TEM-1_	9	19.57%	monobactam; cephalosporin; penam; penem
*bla*_OXA-1_	9	19.57%	phenicol antibiotic
*catB3*	9	19.57%	phenicol antibiotic
*AAC(6′)-Ib10*	9	19.57%	phenicol antibiotic
*QnrS1*	8	17.39%	fluoroquinolone antibiotic
*tet(A)*	7	15.22%	tetracycline antibiotic
*QnrB1*	7	15.22%	fluoroquinolone antibiotic
*dfrA14*	7	15.22%	diaminopyrimidine antibiotic
*CTX-M-15*	7	15.22%	cephalosporin; penam
*AAC(3)-IIe*	7	15.22%	aminoglycoside antibiotic
Others	41	–	–

**Figure 2:**
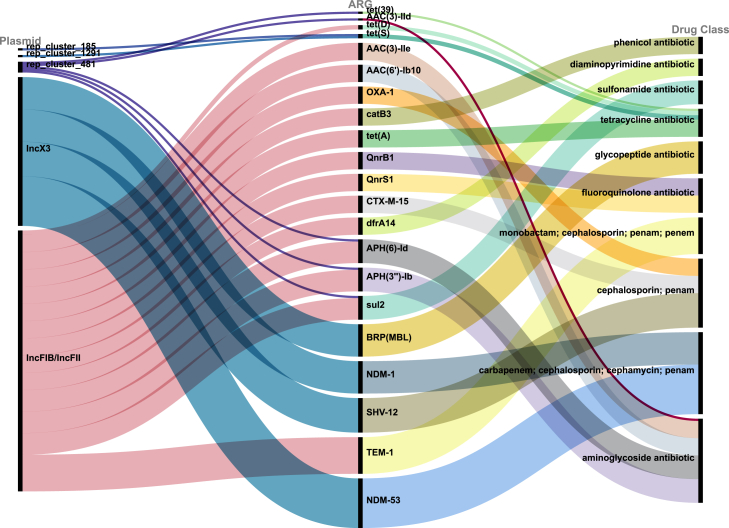
Sankey diagram illustrating the relationship between plasmid types, resistance genes, and associated drug classes The diagram displays the flow from various plasmid types (left) to the identified resistance genes (middle), and further to the corresponding drug classes (right).

Most of ARGs were found in IncFIB/IncFII (121/245, 49.4%) and IncX3 (70/245, 28.6%). All *bla*_NDM-53_, *bla*_TEM-1_, *bla*_SHV-12_, *bla*_NDM-1_, and *BRP(MBL)* genes were detected in InX3-carried conjugants. In contrast, all *tet(A)*, *QnrB1*, *dfrA14*, *CTX-M-15*, *AAC(3)-IIe*, and *sul1* genes were detected in IncFIB/IncFII-carried conjugants (Figure 2).

### The virulence genes carried on plasmids of conjugants

All TARPs isolated from ICU air were uploaded to VFDB for alignment and results showed that 52.17% (24/46) of plasmids of conjugants contained virulence genes such as *htpB*, *ibeB*, and *wbjD*. The gene with the highest detection rate was *htpB* (16/46, 34.78%), followed by *ibeB* (9/46, 19.57%) (Table 5).

**Table 5:** Distribution of virulence genes of plasmids in ICU air of hospitals

Category of VF	Number of positive samples (*n* = 46)	Frequency (%)	Function
*htpB*	16	34.78%	molecular chaperone GroEL
*ibeB*	9	19.57%	copper/silver efflux system outer membrane protein CusC
*wbjD*	1	2.17%	UDP-N-acetylglucosamine 2-epimerase
*tuf*	1	2.17%	elongation factor Tu
*rmlB*	1	2.17%	dTDP-glucose 4,6-dehydratase
*rfaD*	1	2.17%	ADP-L-*glycero*-D-mannoheptose-6-epimerase
*pgi*	1	2.17%	glucose-6-phosphate isomerase
*outG*	1	2.17%	general secretion pathway protein G
*KPHS_35570*	1	2.17%	glucose-1-phosphate thymidylyltransferase
*aslA*	1	2.17%	putative arylsulfatase
*acrB*	1	2.17%	acriflavine resistance protein B

### SNPs analysis of IncX3

**Figure 3:**
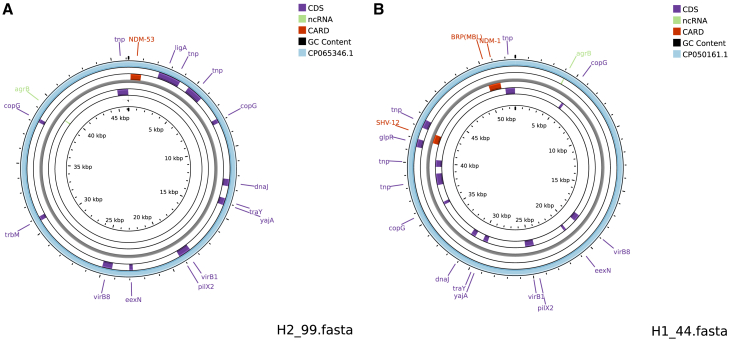
Sequence comparison of IncX3 plasmids Relevant genes are indicated in the outer ring: antibiotic resistance gene is indicated in red, other coding sequences are indicated in purple. The two most inner rings provide a size scale and GC content. The IncX3 plasmids identified in this study are in the most inner colored ring and published plasmids used for comparison are in the most outer colored ring. (A) A plasmid from a thoracic hospital and a plasmid from a *K. pneumoniae* strain (GenBank: CP065346.1). (B) A plasmid from a traditional Chinese medicine hospital and a plasmid from an *E. coli* (GenBank: CP050161.1).

**Figure 4:**
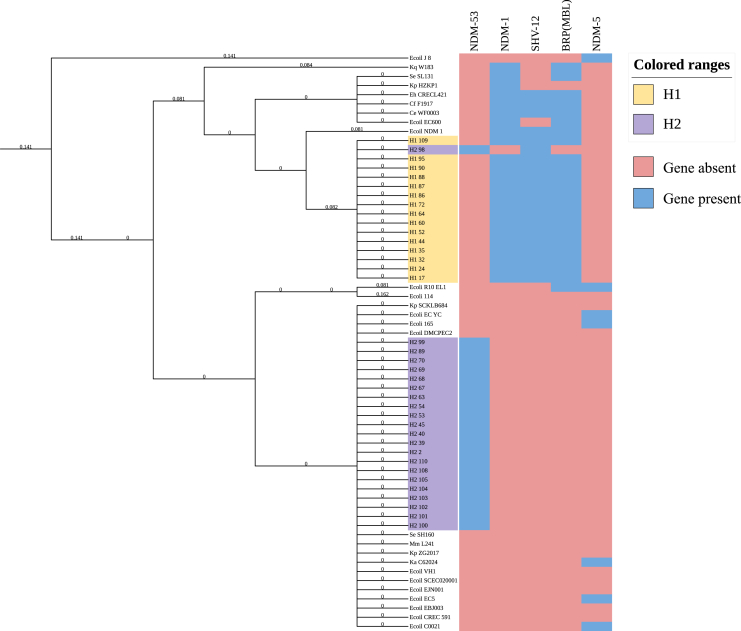
SNP analysis of IncX3 plasmids and heatmap of associated resistance genes SNP analysis of IncX3 were adopted to construct the phylogenetic tree. Thirty-seven were IncX3 extracted in this study, and the rest were other serotypes obtained from GenBank (Table S2). The SNP detection was performed by the snippy pipeline with the *E.coli J8* (GenBank: CP047006.1) as reference. The yellow color was the IncX3 from a traditional Chinese medical hospital, and the purple color represented the IncX3 from a thoracic hospital. The accompanying heatmap illustrates the distribution of associated antibiotic resistance genes across the plasmid variants. Each column represents a specific resistance gene and each row corresponds to a plasmid variant. The blue referred to the presence of the gene in the plasmid and red indicated its absence.

IncX3 was detected in 80.43% (37/46) of the transconjugants obtained from ICU air, originating from two hospitals. To investigate the phylogenetic relationships of the IncX3 plasmids, SNP analysis was conducted on the 37 IncX3 plasmids isolated in this study, along with 26 additional IncX3 sequences retrieved from NCBI (Figures 3 and 4). One cluster, associated with hospital H1, a traditional Chinese medicine hospital, primarily treats chronic conditions such as cerebrovascular diseases. The other cluster is associated with hospital H2, a thoracic hospital that focuses on respiratory diseases. The analysis revealed that IncX3 plasmids originating from the same hospital generally belong to the same phylogenetic lineage, indicating a common evolutionary ancestor. In contrast, plasmids from different hospitals exhibited relatively distinct branches on the phylogenetic tree. The IncX3 plasmids from the traditional Chinese medicine hospital clustered with those isolated from pathogenic bacteria such as *Klebsiella pneumoniae* and *Salmonella enterica*, as well as opportunistic pathogens including *Citrobacter* spp., *Enterobacter cloacae*, and *Escherichia coli*. These reference plasmids primarily derived from clinical samples. While the IncX3 plasmids from the thoracic hospital clustered with those from both pathogenic bacteria (e.g., *K. pneumoniae* and *S. enterica*) and non-pathogenic bacteria (e.g., *E. coli* and *Enterobacter aerogenes*). The sources of these reference plasmids were diverse, including clinical samples, river water, and vegetables.

## Discussion

### Plasmid types and associated resistance genes

The extensive use of antibiotics in clinical settings, particularly in ICUs, has led to the emergence of antibiotic-resistant bacteria and MDR organisms.^20^ ARGs can migrate and disseminate through plasmids and other mobile genetic elements across different ecological environments, resulting in the widespread proliferation of resistance genes and posing significant challenges to human health.^21^ Plasmids are widely recognized as the most common mobile genetic elements carrying ARGs. For instance, carbapenemase resistance genes such as *bla*_NDM-1_, *bla*_NDM-5_, and *bla*_OXA-48_ can be transferred among Enterobacteriaceae through plasmids such as IncF, IncN, and IncX,^22^ leading to the extensive spread of carbapenem-resistant Enterobacteriaceae (CRE). Current research on plasmids primarily focuses on analyzing the resistance and transferability of plasmids after isolating pathogenic bacteria.^23^ However, non-pathogenic bacteria can also harbor resistance plasmids and transfer them to pathogenic strains. Therefore, greater attention should be directed toward the TARPs themselves. As a unique medical environment, ICUs lack comprehensive studies on TARPs present in the air. This study treated entire air samples as donors, without distinguishing between pathogenic and non-pathogenic bacteria, enabling a more thorough investigation of the characteristics of TARPs in ICU air.

This study collected 49 ICU air samples from 13 hospitals, with positive samples distributed across five hospitals, resulting in a conjugation success rate of 20.41% (10/49). Hospitals typically implement multiple layers of strict disinfection measures, including the cleaning of ventilation systems, air conditioning filters, and frequently touched surfaces. Furthermore, most ICU wards utilize laminar flow purification technology to reduce airborne antibiotic-resistant bacteria. Notably, the sampling period coincided with the COVID-19 outbreak, during which hospitals enforced even more stringent environmental hygiene measures, particularly concerning air quality. In addition, many of the samples were taken following routine disinfection procedures in the ICU. Despite these extensive disinfection protocols and study showed that strengthening the implementation of strict terminal disinfection procedures in hospital wards and their surrounding environments significantly helps reduce the risk of hospitalized patients acquiring infections from MDR bacteria,^24^ we still successfully isolated resistance plasmids. This detection of TARPs from air samples in five hospitals highlights that, even with rigorous disinfection practices, hospital air continues to act as a reservoir for resistance genes, underscoring the critical role of ICU air in the dissemination of ARGs. However, due to the uniformity of disinfection practices across the participating hospitals and the relatively small number of positive samples, no statistically significant correlation was observed between disinfection measures and the presence of TARPs. Further research is needed to explore the factors influencing TARPs, including disinfection protocols, patient infections with resistant bacteria in ICUs, and antibiotic usage. Understanding how these factors affect TARPs will be crucial in formulating more detailed infection control strategies for ICUs, helping to prevent HAIs.

Among the 46 TARPs, the most common plasmid type was IncX3 (80.70%, 37/46). This study represents the first identification of transferable IncX3 in the air of hospital ICUs. Previous studies have shown that IncX3 plasmids are among the most widely disseminated plasmids over the past decade.^25^ IncX3 has exhibited significant stability and low adaptation costs in both clinical isolates and transconjugants,^26^ making it less prone to loss during the plasmid transfer process. This characteristic may explain why IncX3 emerged as the most prevalent plasmid type in our study. IncX3 plasmids have been isolated from a wide range of human, animal, and environmental strains globally, especially in China. Notably, two-thirds of these strains originate from clinical settings.^25^ Clinically, IncX3 plasmids are predominantly found in Enterobacteriaceae such as *E. coli* and *K. pneumoniae*.^27^ The isolation of IncX3 from ICU air not only expands the known environmental sources of IncX3 plasmids but also reinforces its high adaptability and resilience. SNP analysis comparing the IncX3 plasmids isolated in this study with 26 sequences downloaded from NCBI revealed high consistency between the IncX3 plasmids found in the air and those present in gastrointestinal pathogens such as *K. pneumoniae* and *Salmonella* spp., as well as in opportunistic pathogens like *Citrobacter* spp. and *Enterobacter cloacae*. This suggests that air may serve as one of the transmission mediums for the resistance plasmid IncX3. Interestingly, IncX3 plasmids from different hospitals occupied distinct phylogenetic branches. Plasmids carrying the ***bla***_NDM-53_ resistance gene were predominantly found in the thoracic hospital, while those harboring ***bla***_NDM-1_, ***bla***_SHV-12_, and *BRP(MBL)* genes primarily originated from the traditional Chinese medicine hospital. This suggests that different hospitals are dominated by distinct IncX3 subtypes. All these plasmids harbored carbapenem resistance genes, indicating the widespread dissemination of carbapenem resistance. The extensive use of β-lactam antibiotics has led to resistance among most clinically significant bacterial pathogens.^28^ The *bla*_SHV-12_ gene is one of the most common genes encoding extended-spectrum β-lactamases (ESBLs) in *Enterobacteriaceae*, conferring resistance to β-lactam antibiotics.^29^ The presence of this gene in transferable plasmids in the air exacerbates its spread. Additionally, the *BRP(MBL)* gene confers resistance to antitumor glycopeptides such as bortezomib, suggesting that TARPs in the air could lead to the emergence of resistant bacteria in oncology wards. The second most frequently isolated plasmid types were IncFIB/IncFII, detected in 18.37% (9/49) of the samples. IncFB and IncFII are often found within the same conjugant, with the FII replicon type is commonly co-occurring with other incompatible replicons within the same plasmid, forming a multireplicon structure.^30^ A total of 14 resistance genes were detected on IncF plasmids, including *bla*_TEM-1_, *APH(3″)-Ib*, *APH(6)-Id*, and *sul2*. Conjugants harboring these plasmids exhibited multidrug resistance, consistent with the previous findings of Johnson et al*.*^31^ The IncF-related family has the potential to significantly contribute to the global dissemination of various resistance genes, making IncF plasmids a critical focus of attention in the study of antibiotic resistance.

Whole-genome data of TARPs was uploaded to the CARD database. The analysis revealed 245 ARGs categorized into 47 subtypes. The most frequent resistance mechanism identified was antibiotic inactivation. The most frequently detected ARG was *bla*_NDM-53_, followed by *bla*_SHV-12_ (16/46) and *BRP(MBL)* (15/46). This finding contrasts with a previous study conducted in a comprehensive tertiary hospital, which reported dominance of *bacA*, acetyltransferase (*cat*), and *lnuA* in the air.^32^ Possible reason for this inconsistency with prior studies may be differences in antibiotic usage patterns or environmental backgrounds. ICU patients often have more severe illnesses, resulting in the use of different antibiotics compared to outpatient settings, which may influence the abundance of resistance genes. Additionally, previous literature primarily focused on the totality of resistance genes, including those located on chromosomes and plasmids, whereas this study specifically examined TARPs, which could also account for variations in the abundance of resistance genes. In this study, *bla*_NDM-53_, *bla*_SHV-12_, *bla*_NDM-1_, *BRP(MBL)* are all carried by IncX3 plasmids. This is consistent with previous research showing that IncX3 is a key vector for the *bla*_NDM_ gene.^33^ However, in contrast to earlier studies where *bla*_NDM-5_ and *bla*_NDM-1_ were the most commonly observed variants,^33^ the most common variants are *bla*_NDM-53_ and *bla*_NDM-1_ in this study. We identified a novel variant within the New Delhi metallo-β-lactamase (NDM) family, *bla*_NDM-53_, which primarily exhibits resistance to carbapenem antibiotics. The *bla*_NDM-1_ and *bla*_NDM-53_ were the first discovered subtype of carbapenem ARG isolated from ICU air in hospital. The presence of *bla*_NDM-1_ and *bla*_NDM-53_ on transferable plasmids in hospital air suggests the possibility of airborne transmission of these genes, highlighting the need for further investigation. It is worth noting that carbapenems are considered last-resort antibiotics for the treatment of infections caused by MDR Gram-negative bacteria. IncX3 subgroup is primarily responsible for the dissemination of ARGs for clinically relevant first-line and last-resort (carbapenems) antibiotics.^29^ Its high transferability in the air, strong association with carbapenem resistance genes, and the emergence of novel variants underscore the urgent need to address the growing threat of carbapenem resistance, particularly through airborne transmission.

### Phenotype of the transconjugants

In this study, the recipient strain *E. coli* CV601 initially exhibited resistance only to rifampicin and kanamycin. Following conjugation experiments, we conducted drug susceptibility testing on 57 hospital air-derived transconjugants, revealing varying degrees of resistance to all 17 antibiotics tested. Notably, 96.49% of the transconjugants exhibited resistance to at least four antibiotics, indicating a severe prevalence of multidrug resistance. This included six transconjugants resistant to 15 antibiotics, 13 transconjugants resistant to 14 antibiotics, three transconjugants resistant to 12 antibiotics, and 12 transconjugants resistant to 11 antibiotics. These findings suggest that during the plasmid transfer process, multiple resistance genes were simultaneously transferred into the recipient bacteria, endowing them with inherent resistance characteristics and thereby enhancing their overall resistance. This level of horizontal gene transfer underscores the significant role that airborne plasmids play in the transfer of resistance, particularly concerning the emergence of unexplained new resistant bacteria. Additionally, the transconjugants displayed the highest resistance to β-lactam antibiotics such as CTX, CAZ, CFT, and AMP. In contrast, they were sensitive to AMK, TGC, TET, CHL, SXT, GEN, and STR, with TGC demonstrating particularly high sensitivity. These results are consistent with Liu Zhiwu’s findings, which indicated that the resistance levels to β-lactam antibiotics ranged from 90% to 100%, while the resistance to AMK was comparatively lower.^34^ According to the CHINET 2022 national bacterial resistance monitoring report, the resistance of intestinal pathogens to a range of commonly used antibiotics, including aminoglycosides, carbapenems, tetracyclines, third-generation cephalosporins, and sulfonamides, is increasingly severe, particularly among Gram-negative bacteria resistant to carbapenems, which remain at a high detection rate. Moreover, the data indicate that certain major resistant Enterobacteriaceae still maintain relatively high sensitivity to TGC and AMK, consistent with the findings of this study regarding airborne samples.^35^

### Conclusion

TARPs are present in the air of ICUs. The predominant transferable plasmid type is IncX3, with *bla*_NDM_ being the primary ARG. The *bla*_NDM-1_ and *bla*_NDM-53_ are the first discovered subtype of carbapenem ARG isolated from ICU air in hospital. Our findings suggest that air in ICUs may serve as a critical pathway for the spread of MDR genes within hospital environments.

### Limitations of the study

There are limitations in present study. First, the donor bacteria of the TARPs could not be identified. However, the focus should be on the TARPs themselves, regardless of whether they originate from pathogenic or non-pathogenic bacteria. Therefore, this limitation does not compromise our primary focus on TARPs. Second, the antibiotic-resistant plasmids isolated in this study were successfully conjugated under selective pressure from antibiotics. However, there is currently insufficient evidence to support whether antibiotics in the hospital ICU environment may lead to the transfer of plasmids. Last, the number of positive samples was limited. Nonetheless, this study revealed the presence of transferable plasmids in the air and identified MDR plasmids. More importantly, the study first discovered the presence of *bla*_NDM-1_ and *bla*_NDM-53_ in the air of ICU.

## Resource availability

### Lead contact

Further information and requests for resources and reagents should be directed to and will be fulfilled by the lead contact, Peng He (549478426@qq.com).

### Materials availability

This study did not generate unique reagents.

### Data and code availability

• Plasmid sequence data supporting this study are available at NCBI under accession numbers PQ872731–PQ872852.
• This study did not generate original code.
• Any additional information required to reanalyze the data reported in this paper is available from the lead contact upon request.

## Acknowledgments

This work was supported by grants from the 10.13039/501100001809National Natural Science Foundation of China (82103800), the Key Project of Medicine Discipline of Guangzhou (No. 2025-2027-11, No. 2025-2027-12) and 10.13039/501100010256Guangzhou Municipal Science and Technology Project (202102080111, 202201010800, 2023A03J0933, and 2024A03J0423).

## Author contributions

K.Z., methodology, formal analysis, investigation, writing – original draft, and writing – review & editing. X. Zhou, methodology, formal analysis, investigation, data curation, and visualization. Xu Zhang, investigation, resources, writing – original draft, and visualization. N.H., investigation and resources. Z.Z., investigation and resources. Xinqiang Zhang: investigation and resources. Y.z., investigation and resources. J.L., investigation and resources. F.Y., investigation and data curation. Y.L., investigation, resources, and funding acquisition. P.Q., methodology, conceptualization, supervision, project administration, and funding acquisition. X.W., conceptualization, supervision, project administration, and funding acquisition. P.H., methodology, software, validation, formal analysis, investigation, resources, data curation, writing – original draft, writing – review & editing, visualization, and funding acquisition.

## Declaration of interests

The authors declare no competing interests.

## STAR★Methods

### Key resources table

**Table:** 

REAGENT or RESOURCE	SOURCE	IDENTIFIER
**Antibodies**		

Kanamycin	Solvay	K8020
Rifampicin	Solvay	R8011
Levofloxacin	Solvay	L8730
Gentamicin	Solvay	G8170
Meropenem	Solvay	IM0120
Cefotiam	Solvay	C8240
Ceftazidime	Solvay	IC4140
Clarithromycin	Solvay	C9490
Polymyxin B	Solvay	P8350
Ciprofloxacin	Solvay	C9710
Chloramphenicol	Solvay	C8050
Tigecycline	Solvay	T6990-1g

**Bacterial and virus strains**		

*Escherichia coli* CV601	Professor Zhongdao Wu’s laboratory	–

**Critical commercial assays**		

TIANpure Midi Plasmid Kit	TIANGEN BIOTECH (BEIJING) CO.,LTD.	GDP107-02

**Deposited data**		

Analyzed data	This paper	Genbank: PQ872731 - PQ872852
Plasmid sequence with Accession Number CP137186.1	National Center for Biotechnology Information (NCBI)	https://www.ncbi.nlm.nih.gov/nuccore/CP137186.1
Plasmid sequence with Accession Number CP133344.1	National Center for Biotechnology Information (NCBI)	https://www.ncbi.nlm.nih.gov/nuccore/CP133344.1
Plasmid sequence with Accession Number CP050161.1	National Center for Biotechnology Information (NCBI)	https://www.ncbi.nlm.nih.gov/nuccore/CP050161.1
Plasmid sequence with Accession Number MH105052.2	National Center for Biotechnology Information (NCBI)	https://www.ncbi.nlm.nih.gov/nuccore/MH105052.2
Plasmid sequence with Accession Number CP139934.1	National Center for Biotechnology Information (NCBI)	https://www.ncbi.nlm.nih.gov/nuccore/CP139934.1
Plasmid sequence with Accession Number CP163020.1	National Center for Biotechnology Information (NCBI)	https://www.ncbi.nlm.nih.gov/nuccore/CP163020.1
Plasmid sequence with Accession Number MH105050.1	National Center for Biotechnology Information (NCBI)	https://www.ncbi.nlm.nih.gov/nuccore/MH105050.1
Plasmid sequence with Accession Number CP149133.1	National Center for Biotechnology Information (NCBI)	https://www.ncbi.nlm.nih.gov/nuccore/CP149133.1
Plasmid sequence with Accession Number CP047006.1	National Center for Biotechnology Information (NCBI)	https://www.ncbi.nlm.nih.gov/nuccore/CP047006.1
Plasmid sequence with Accession Number CP020512.1	National Center for Biotechnology Information (NCBI)	https://www.ncbi.nlm.nih.gov/nuccore/CP020512.1
Plasmid sequence with Accession Number CP169200.1	National Center for Biotechnology Information (NCBI)	https://www.ncbi.nlm.nih.gov/nuccore/CP169200.1
Plasmid sequence with Accession Number CP024825.1	National Center for Biotechnology Information (NCBI)	https://www.ncbi.nlm.nih.gov/nuccore/CP024825.1
Plasmid sequence with Accession Number MW415440.1	National Center for Biotechnology Information (NCBI)	https://www.ncbi.nlm.nih.gov/nuccore/MW415440.1
Plasmid sequence with Accession Number CP086335.1	National Center for Biotechnology Information (NCBI)	https://www.ncbi.nlm.nih.gov/nuccore/CP086335.1
Plasmid sequence with Accession Number CP060972.1	National Center for Biotechnology Information (NCBI)	https://www.ncbi.nlm.nih.gov/nuccore/CP060972.1
Plasmid sequence with Accession Number CP084538.1	National Center for Biotechnology Information (NCBI)	https://www.ncbi.nlm.nih.gov/nuccore/CP084538.1
Plasmid sequence with Accession Number CP086339.1	National Center for Biotechnology Information (NCBI)	https://www.ncbi.nlm.nih.gov/nuccore/CP086339.1
Plasmid sequence with Accession Number CP032424.1	National Center for Biotechnology Information (NCBI)	https://www.ncbi.nlm.nih.gov/nuccore/CP032424.1
Plasmid sequence with Accession Number CP028705.1	National Center for Biotechnology Information (NCBI)	https://www.ncbi.nlm.nih.gov/nuccore/CP028705.1
Plasmid sequence with Accession Number CP139378.1	National Center for Biotechnology Information (NCBI)	https://www.ncbi.nlm.nih.gov/nuccore/CP139378.1
Plasmid sequence with Accession Number MH781720.1	National Center for Biotechnology Information (NCBI)	https://www.ncbi.nlm.nih.gov/nuccore/MH781720.1
Plasmid sequence with Accession Number CP065346.1	National Center for Biotechnology Information (NCBI)	https://www.ncbi.nlm.nih.gov/nuccore/CP065346.1
Plasmid sequence with Accession Number CP033057.1	National Center for Biotechnology Information (NCBI)	https://www.ncbi.nlm.nih.gov/nuccore/CP033057.1
Plasmid sequence with Accession Number CP053295.1	National Center for Biotechnology Information (NCBI)	https://www.ncbi.nlm.nih.gov/nuccore/CP053295.1
Plasmid sequence with Accession Number OR095749.1	National Center for Biotechnology Information (NCBI)	https://www.ncbi.nlm.nih.gov/nuccore/OR095749.1

**Software and algorithms**		

Trimmomatic	Bolger et al.^36^	https://github.com/usadellab/Trimmomatic
SPAdes (version 3.13.1)	Bankevich et al.^37^	https://github.com/lh3/bwa
Unicycler	Wick et al.^38^	https://github.com/rrwick/Unicycler
Burrows-Wheeler Aligner	LI et al.^39^	https://bio-bwa.sourceforge.net/
CARD (The Comprehensive Antibiotic Resistance Database)	Jia et al.^40^	https://card.mcmaster.ca/
VFDB (Virulence Factors of Pathogenic Bacteria)	Chen et al.^41^	http://www.mgc.ac.cn/VFDB/
PlasmidFinder	Carattoli et al.^42^	http://www.cge.cbs.dtu.dk/services/PlasmidFinder/
Mob_recon	Robertson et al.^43^	https://github.com/phac-nml/mob-suite
Proksee	Grant et al.^44^	https://proksee.ca
Snippy pipeline (V4.5.0)	Seemann et al.^45^	https://github.com/tseemann/snippy

### Experimental model and study participant details

#### Microbial strains

Environmental Bacterial Donors: Bacteria were passively collected from airborne particulate matter in hospital ICUs, serving as potential plasmid donors in conjugation experiments.

Recipient Strain: *Escherichia coli* CV601 was used as the recipient strain in conjugation assays.

Genetic Characteristics of CV601: CV601 is resistant to rifampin and kanamycin and carries a GFP (green fluorescent protein) tag, facilitating selection and visualization in conjugation experiments.

#### Experimental conditions

Airborne Bacteria Collection: ICU airborne bacterial samples were collected using the AirPort MD8 device.

Bacterial Growth Conditions:

Donor bacteria: Cultured in 1/10 TSB (Tryptic Soy Broth) at 28°C, 150 rpm, for 24 hours in a shaking incubator.

Recipient strain (CV601): Cultured in LB broth at 37°C under static conditions for 24 hours, with selective antibiotics (rifampin and kanamycin) as needed.

Conjugation Experiment: Conjugation assays were performed to assess the transferability of plasmids from airborne bacteria to *E. coli* CV601.

#### Institutional permission and oversight

This study did not involve human or animal subjects and did not require institutional ethical approval.

Airborne bacteria were passively collected from ICU environments without patient interaction.

#### Study limitations

Donor bacteria were derived from ICU air samples and were not isolated as individual species prior to conjugation assays.

Environmental factors may influence plasmid transfer efficiency, which should be considered when interpreting the results.

### Method details

#### Sample collection

The hospitals selected for this study were identified through a collaboration with multiple healthcare institutions in Guangzhou, the third-largest city in China, encompassing both general and specialized hospitals. In total, samples were collected from 13 hospitals distributed in 11 districts (selected medical institutions across 11 districts in Guangzhou), comprising 12 tertiary hospitals and 1 primary hospital. Among the tertiary hospitals, 4 were specialized in specific fields, while the remaining 8 were general tertiary hospitals.

Air samples were collected using the AirPortMD8 device, with four machines deployed in each ward for one hour. Sample collection was standardized across hospitals, occurring daily between 10:00 AM and 11:00 AM to minimize the potential impact of diurnal variations. Four membranes were obtained and subsequently combined into a single sample for further experiments. The collected samples were directly used as donors, with *E. coli* CV601 as the recipient strain for conjugation experiments.

#### Conjugation

Transfer of antibiotic resistance to *E. coli* CV601 recipients was attempted by filter-mating experiments, as previously described.^46^ The strain *E. coli* CV601, characterized by the expression of the *gfp* gene (green fluorescent protein) and kanamycin (KAN) and rifampicin (RIF) resistance as selection markers.^47^ Transconjugants were selected on Mueller-Hinton (MH) agar plates containing kanamycin (50μg/mL), rifampicin (50μg/mL) and one of the ten primary screening antibiotics including levofloxacin (2μg/mL), gentamicin (16μg/mL), meropenem (4μg/mL), cefotiam (24μg/mL), ceftazidime (16μg/mL), clarithromycin (32μg/mL), polymyxin B (4μg/mL), ciprofloxacin (1μg/mL), chloramphenicol (32μg/mL) and tigecycline (4μg/mL). Transconjugants were confirmed by their green fluorescence. Subsequently, the green fluorescent bacteria were inoculated onto triple-antibiotic agar plates for purification. After confirming growth and the presence of green fluorescence, the strains were preserved.

#### Determination of antibiotic susceptibility

Antimicrobial susceptibility testing was performed using a microdilution method based on the Fosun Diagnostics antibiotic susceptibility panels. Antibiotic resistance was interpreted according to the Clinical Laboratory Standards Institute (CLSI) guidelines (M100-S30). For antibiotics not included in this standard, the EUCAST guidelines were used.

The MICs of 17 antibiotics against conjugants were determined using the broth microdilution method. A higher MIC value indicates lower bacterial sensitivity to the antibiotic. Minimum Inhibitory Concentrations (MICs) were determined by inoculating the wells with a standardized bacterial suspension (0.5 McFarland standard) prepared in Mueller-Hinton broth. The plates were incubated in an aerobic environment at 35 ± 2°C for 16–20 hours. All tests were performed in triplicate to ensure reproducibility. The reported MICs reflect the median of three independent experiments, with an acceptable variability of ±1 dilution.

Antibiotics tested included clarithromycin (CLA), ampicillin (AMP), ertapenem (ETP), meropenem (MEM), ceftazidime (CAZ), cefotaxime (CTX), cefotiam (CFT), amikacin (AMK), gentamicin (GEN), streptomycin (STR), polymyxin B (PB), chloramphenicol (CHL), cotrimoxazole (SXT), ciprofloxacin (CIP), nalidixic acid (NAL), tetracycline (TET), and tigecycline (TGC). Results were categorized as Sensitive (S), Intermediate (I), or Resistant (R).

#### Extraction of plasmid DNA and whole-genome sequencing

Plasmid DNA was extracted from the recipient bacteria that successfully conjugated using the TIANpure Midi Plasmid Kit (TIANGEN BIOTECH, Beijing, China). The concentration and purity of the plasmids were assessed using a Qubit fluorimeter (Thermo Fisher, US) and NanoDrop1000. Plasmids with a purity (A260/280 ≥ 2) and concentration (≥ 50μg/mL) were subjected to library preparation. Sequencing was performed using the MGISP-100 DNA Sequencing Library Preparation System (MGI Tech Co., Ltd.) for second-generation sequencing and the nanopore (Oxford Nanopore Technologies) for third-generation sequencing.

#### Bioinformatics analysis

Sequencing results were analyzed to determine the presence of TARPs, plasmid types, and the distribution of resistance genes on the plasmids. Quality control was performed using Trimmomatic, and assembly was conducted with SPAdes (v3.13.1) using default parameters, along with hybrid assembly performed using Unicycler. BWA for alignment and analysis of the nucleotide sequence of the recipient bacterium *E. coli* CV601, with removal of the recipient bacterium sequence in the conjugants. Annotation and functional identification of resistance genes and virulence genes were performed by comparison with the Comprehensive Antibiotic Research Database (CARD) and Virulence Factors Database (VFDB) databases, while categories of plasmids were annotated using PlasmidFinder and Mob_recon. Genes with less than 75% homology and 60% coverage were excluded. The Proksee online platform was used to generate plasmid circular maps. The SNP analysis was conducted with Snippy pipeline (v4.5.0).

### Quantification and statistical analysis

A chi-square test was performed to assess the association between the presence of antibiotic resistance plasmids in ICU air and various hospital-related factors, including ICU bed count, disinfection measures and frequencies for air, surfaces, and floors, as well as ICU and patient types. Statistical analysis was conducted using IBM SPSS Statistics 26 (IBM, USA). The results showed no significant correlation (*p* > 0.05). Since no additional statistical analyses were performed, no related figures or tables were included in the manuscript.
