# Supplementary material for: Characterization of transferable antibiotic resistance plasmids in airborne particulate matter from ICU environments
Source: iScience. 2025 Mar 20;28(5):112254. doi: 10.1016/j.isci.2025.112254 (PMC12052693; doi:10.1016/j.isci.2025.112254)
Supplement: Document S1. Tables S1 and S2 [file mmc1.pdf]

iScience, Volume *28*

Supplemental information

Characterization of transferable antibiotic resistance plasmids in airborne particulate matter from ICU environments

Kexing Zhang, Xumei Zhou, Xu Zhang, Na Huang, Zhengyang Zhao, Xinqiang Zhang, Yong zhou, Juntao Li, Fangyi Yu, Yuan Liu, Pengzhe Qin, Xinwei Wu, and Peng He

Table S 1.ICU Sampling Points and Their Disinfection Measures

Sampling Point	Number of Beds	ICU Type	Main Patient Type	Air Disinfection Measures	Surface Disinfection and Frequency	Floor Disinfection and Frequency
H1	15	General	General	Laminar flow	Chlorine disinfectant, three times a day (Kangbili disinfecting wipes)	Chlorine disinfectant, twice a day
H2	30	General	General	Laminar flow	Chlorine disinfectant, twice each time	Chlorine disinfectant, twice a day
H3	9	General	General	Laminar flow	Chlorine disinfectant, three times a day (Jielijia surface disinfecting wipes)	Chlorine disinfectant, twice a day
H4	18	General	General	Laminar flow	Chlorine disinfectant, three times a day	Chlorine disinfectant, three times a day
H5	20	General	General	Laminar flow	Chlorine disinfectant, three times a day	Chlorine disinfectant, twice each time
H6	12	General	General	Laminar flow	Chlorine disinfectant, twice each time	Chlorine disinfectant, twice a day
H7	9	General	Various critically ill patients related to psychiatry	Laminar flow	Chlorine disinfectant, twice each time	Chlorine disinfectant, twice a day
H8	12	General	Oncology critically	Laminar flow	Chlorine disinfectant,	Chlorine disinfectant,

			ill patients		twice each time	twice a day
H9	42	PICU	Pediatric	Laminar flow	Chlorine disinfectant, twice each time	Chlorine disinfectant, twice a day
H10	27	General	General	Laminar flow	Chlorine disinfectant, twice each time	Chlorine disinfectant, twice a day
H11	9	PICU	Pediatric	Laminar flow	Chlorine disinfectant, twice each time	Chlorine disinfectant, twice a day
H12	20	General	General	Laminar flow	Chlorine disinfectant, three times a day	Chlorine disinfectant, three times a day
H13	42	PICU	Pediatric	Laminar flow	Chlorine disinfectant, twice each time	Chlorine disinfectant, twice a day

Table S 2.Summary of Sequences, Isolation Sources, and GenBank Accession Numbers

Sequence Name	Isolation Source	GenBank Accession Number
Cf F1917	*Citrobacter freundii* F1917 Genome sequencing and assembly	CP137186.1
Eh CRECL421	hospital sample	CP133344.1
Ecoil NDM-1	Carbapenemase-producing *Enterobacteriaceae* isolated from hospitals, animals and healthy people.	CP050161.1
Ecoil EC600		MH105052.2
Kq HZKP1	Pathogen: clinical or host-associated sample of *Klebsiella pneumoniae* from blood	CP139934.1
Kq W183	Pathogen: clinical or host-associated sample from *Klebsiella quasipneumoniae*	CP163020.1

Se SL131	fecal sample	MH105050.1
Ce WF0003	patient's ascites	CP149133.1
Ecoil J8	Microbe sample(intestinal and liver) from *Escherichia coli*	CP047006.1
Ecoil 165	Pathogen: clinical or host-associated sample from *Escherichia coli*	CP020512.1
Ecoil C0021	Microbe sample from *Escherichia coli*	CP169200.1
Ecoil CREC-591	Pathogen: clinical or host-associated sample from *Escherichia coli*	CP024825.1
Ecoil DMCPEC2	MIGS Cultured Bacterial/Archaeal sample from *Escherichia coli*	MW415440.1
Ecoil EBJ003	Pathogen: clinical or host-associated sample from *Escherichia coli*	CP086335.1
Ecoil EC5	Pathogen: clinical or host-associated sample from *Escherichia coli*	CP060972.1
Ecoil EC-YC	MIGS Cultured Bacterial/Archaeal sample from *Escherichia coli*	CP084538.1
Ecoil EJN001	Pathogen: clinical or host-associated sample from *Escherichia coli*	CP086339.1
Ecoil SCEC020001	Microbe sample from *Escherichia coli*	CP032424.1
Ecoil VH1	Pathogen: environmental/food/other sample from *Escherichia coli*	CP028705.1
Ka C62024	*Klebsiella aerogenes* host-associated isolate C62024	CP139378.1
Kp SCKLB684	RefSeq.	MH781720.1
Kp ZG2017	Microbe sample(river) from *Klebsiella pneumoniae*	CP065346.1
Mm L241	Pathogen: clinical or host-associated sample from faeces	CP033057.1
Se SH160	Beta-lactamase sample from *Salmonella enterica* subsp. enterica serovar Typhimurium	CP053295.1
Ecoil R10-EL1	MIGS Cultured Bacterial/Archaeal sample from *Escherichia coli*	OR095749.1
Ecoil 114	Microbe sample from *Escherichia coli*	CP087572.1
